# Cognitive impairment categorized in community-dwelling older adults with and without dementia using in-home sensors that recognise activities of daily living

**DOI:** 10.1038/srep42084

**Published:** 2017-02-08

**Authors:** Prabitha Urwyler, Reto Stucki, Luca Rampa, René Müri, Urs P Mosimann, Tobias Nef

**Affiliations:** 1 Gerontechnology & Rehabilitation Group, University of Bern, Bern, Switzerland; 2 ARTORG Center for Biomedical Engineering Research, University of Bern, Bern, Switzerland; 3 University Hospital of Old Age Psychiatry, University of Bern, Anna-Seiler-Haus,Bern, Switzerland; 4 University Neurorehabilitation Clinics, Department of Neurology, Inselspital, and University of Bern, Anna-Seiler-Haus,, Bern, Switzerland

## Abstract

Cognitive impairment due to dementia decreases functionality in Activities of Daily Living (ADL). Its assessment is useful to identify care needs, risks and monitor disease progression. This study investigates differences in ADL pattern-performance between dementia patients and healthy controls using unobtrusive sensors. Around 9,600 person-hours of activity data were collected from the home of ten dementia patients and ten healthy controls using a wireless-unobtrusive sensors and analysed to detect ADL. Recognised ADL were visualized using activity maps, the heterogeneity and accuracy to discriminate patients from healthy were analysed. Activity maps of dementia patients reveal unorganised behaviour patterns and heterogeneity differed significantly between the healthy and diseased. The discriminating accuracy increases with observation duration (0.95 for 20 days). Unobtrusive sensors quantify ADL-relevant behaviour, useful to uncover the effect of cognitive impairment, to quantify ADL-relevant changes in the course of dementia and to measure outcomes of anti-dementia treatments.

Cognitive impairment due to Alzheimer’s disease and other forms of dementia affect patient’s ability to maintain activities of daily living (ADL)^1^. This has severe implications on patient’s independence and quality of life^2^. Impaired ADL function is also the main reason for increased need for care or institutionalization^3^.

ADL refers to self-care tasks, comprising of activities performed on a daily basis^4^,^5^ that a person needs to perform autonomously. ADL are classified in two groups; those involving core tasks of everyday life such as eating, dressing and bathing, termed as basic ADL^4^,^5^, and those involving complicated higher-level tasks involving interactions with “instruments” such as preparing meals, managing finances and using the telephone, termed as Instrumental ADL (IADL)^3^. To live safe and independently at home a person needs to perform ADL from both groups, reliable and autonomously. Though both, basic ADL and IADL are important for safe and independent living, competence in IADL is necessary criteria for living independently in community-dwelling setup^3^,^6^. In this manuscript we use ADL generally, to refer to both groups. ADL are important predictors of quality of life^7^,^8^ and are assessed by clinicians to benchmark the physical and cognitive abilities of patients^3^, to determine care needs, identify risks in daily living and monitor disease progression or the effect of anti-dementia treatment^1^. Traditionally, ADL are assessed using self-rated patient questionnaires or informant based questionnaires (e.g. Katz Activities of Daily Living^3^, Stanford Health Assessment Questionnaire^9^ and the Barthel ADL Index^10^) or by direct observation of the patient when doing a task. Task observations are time-consuming and prone to transfer errors from lab to reality. A downside of questionnaires lies in their reliance on subjective ratings of participant or relatives and, therefore, subject to bias and errors linked to cognitive impairment or lack of insight into impairments^11^. Moreover, as many patients live alone and are supported for a few hours a week, it is difficult to get a reliable and comprehensive clinical picture of the patient’s ADL status.

Sensor-based technologies for quantifying ADL can add new dimensions to existing clinical assessment. Based on constant monitoring, it can help in earlier disease and risk detection^12^,^13^, delay in institutionalization by adjusting care to the patient’s needs and thus lower cost of medical care^14^,^15^. Such sensor-based recognition systems belong to the field of assistive technologies which aim at prolonging independent living in one’s own home^16^. The most important components of sensor-based recognition systems are the sensors which collect the data in the patient’s environment (ambient sensors) or directly on the patient (body-mounted sensors), the wireless transmission protocol responsible for transferring collected data to the receptor unit, and the central computing unit with necessary algorithms for data interpretation and analysis.

Recognizing ADL in home settings using sensor systems has been well-reported in literature^17^,^18^,^19^,^20^,^21^ classifying them into five main types of monitoring technologies: passive infrared motion sensors (PIR), body-mounted sensors, pressure sensors, video monitoring, and multicomponent sensors. Of these, ambient sensor systems^21^ and body-mounted systems^22^ are widely reported for recognizing ADL; while only few studies have tried to combine data from both or multiple sensors^17^. Ambient sensors such as PIR sensors are sensitive to body emitted infrared light and detect presence of residents in rooms, thus allowing recognition of patterns in daily activity^19^,^21^, while body-mounted sensors systems have the ability to measure activity and mobility directly on the patient’s body^22^. Several authors suggest that the usability and acceptance of ambient sensors is better compared to body-mounted systems because patients are not in direct contact with the sensors^20^.

The use of sensor-based measurement generates large amounts of data, which requires recognition techniques to infer an activity. ADL recognition from ambient data is usually done using training data or prior knowledge based algorithms such as probabilistic based^23^,^24^, rule based^21^,^25^, Naïve Bayes^24^, K-Means clustering^25^ and Random Forest^26^. Another general approach to activity recognition is to design and use machine learning methods to map a sequence of sensor events to a corresponding activity label^19^,^24^.

In this study, we used a wireless unobtrusive (ambient, non-wearable, non-camera based and not requiring any interaction with the user) sensor network to capture ambient environmental data in the home of ten dementia patients and ten age-matched healthy controls for twenty consecutive days. To date, sensor-based ADL recognition studies generally include healthy elderly subjects in home setups or living lab setups, while the scope of our trial includes ten dementia patients with moderate to severe dementia living in a community setup. Qualitatively, the recognized ADL are visualized using colour coded ambulatograms for the cumulative measurement duration, to generate activity maps^27^. Inspired by the Poincaré plot (PP)^28^,^29^ technique, we quantified ADL performance using PP, in addition to the data analysis methods to qualitatively classify and recognize ADL. Receiver Operating Characteristic (ROC)^30^ were used to analyse discriminatory capability of the ADL performance and classification. The primary aim of this study is to investigate the extent of difference in ADL (both basic ADL and IADL) patterns between the healthy controls and dementia patients and to investigate if the difference in ADL can be used to classify the subjects into the two groups. The secondary aim of the study was to investigate the influence of the measurement duration on the classification performance. We hypothesize that irregularities in ADL and dysfunctions in daily routine can be recognized and quantified with the aid of an unobtrusive sensor-based recognition system. In addition, we hypothesize that a non-intrusive system, which does not use body-mounted sensors, avoids video-based imaging and microphone recordings, would be better suited for use in dementia patients due to less patient compliance.

## Results

### Difference of ADL patterns between dementia patients and healthy age-matched control

**Figure 1:**
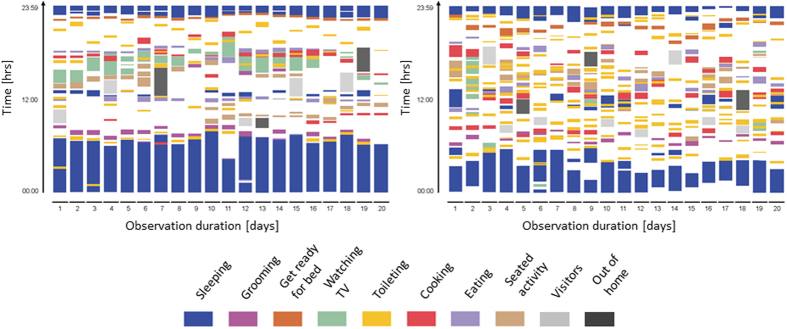
Activity maps of a healthy control (left) and a dementia patient (right) visualized from data measured continuously for 20 consecutive days.

The classification process recognized 4562 ADL in total for both patients and healthy controls. Table 1 shows the apportionment of the determined ADL in detail. Although the sensitivity and specificity of the circadian activity rhythm (CAR) classifier^27^ used to classify ADL is high (94.36% and 98.17% respectively)^27^, it is possible that specific measurement errors exist which could confound the results and analysis presented here. The number of recognized ADL did not vary much between the healthy controls and the dementia patients. However, the continuity and regularity of the ADL performed showed a difference as seen in the activity map (Fig. 1).

Figure 1 shows a comparison of the activity map of a healthy subject (Age = 87 years, female, MMSE = 28) (left) and an Alzheimer patient (Age = 82 years, female, MMSE = 13) (right) for the measurement duration of 20 days. The activity map easily points out the main periods of activity and inactivity and temporal frequencies of the activities.

**Figure 2:**
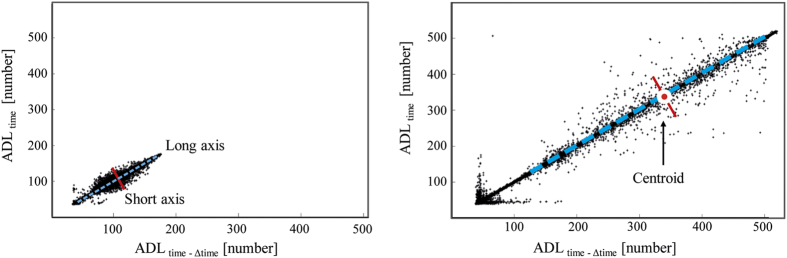
Poincaré Plot of a healthy control (Age = 79 years, female, MMSE = 29) (left) and an Alzheimer patient (Age = 84 years, female, MMSE = 20) (right) from all activity of daily living (ADL) related datasets of 20 consecutive days (Δtime = 24 hours). The blue dotted line indicates the long axis, the red line indicates the short axis. The centroid corresponds to the point where the long axis intersects the short axis.

An example of the PP for a healthy subject and an Alzheimer patient for the complete measurement duration is shown in Fig. 2. The PP descriptors such as long axis, short axis and centroid are also marked in Fig. 2. On quantifying the variability in ADL performance over 20 days using PP centroid, a significant difference in performance of most of the ADL (Sleeping, Getting ready for bed, watching TV, Toileting, Cooking, Seating Activities) was found between healthy controls and dementia patients as shown in Table 1. The heterogeneity in ADL performance of the dementia patients was higher than the healthy controls for all ADL.

### Classification performance and the influence of observation duration

**Figure 3:**
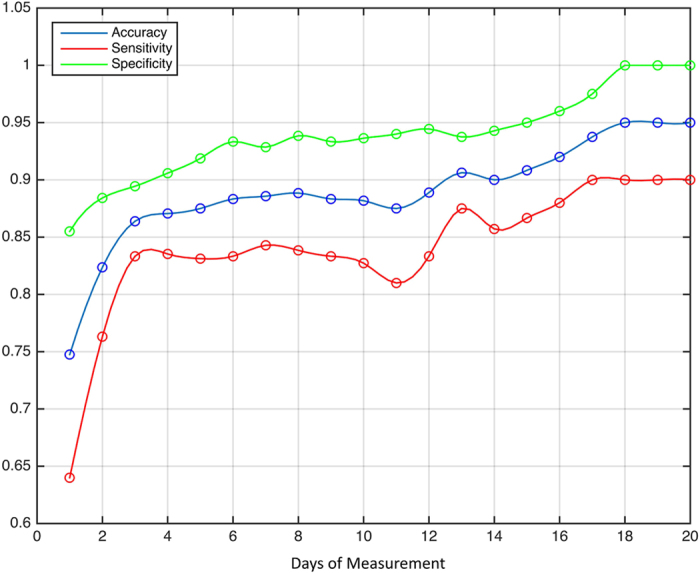
Discriminating ability between healthy controls and dementia patients in dependence of measurement duration, where days of measurement refer to 20 consecutive days.

With the aid of the ROC^30^ (w.r.t. PP centroids), an optimal cut-off value of 69 was deduced as a discriminative power for distinction between the healthy subjects and the dementia patients (Fig. 3). As seen in Fig. 3, the accuracy, sensitivity and specificity of the ADL classification and recognition increases with increasing duration of measurement. After 20 consecutive days of measurement, an accuracy of 0.95, sensitivity of 0.90, and specificity of 1.00 was reached with a starting accuracy of 0.75, sensitivity of 0.64, and specificity of 0.85 on day 1. On an average, the accuracy gains 1.01%, the sensitivity gains 1.30% and the specificity gains 0.72% with every additional day of measurement.

## Discussion

In this observational study, we investigated ADL behaviour patterns and ADL performances of ten healthy controls and ten patients with dementia from data collected using an unobtrusive wireless sensor network in the home of the participants for 20 consecutive days. As hypothesized, the irregularities in ADL performance, as well as dysfunctions in daily routine can be recognized and quantified using unobtrusive sensor-based recognition system and activity map based visualization techniques. Differences in ADL patterns were significant between healthy controls and patients and the accuracy of the classification performance increased with the duration of measurement. There were no drop-outs from the recruitment group and infers the acceptance of the unobtrusive sensor system by healthy older adults and dementia patients. Such sensor-based recognition systems can be vital for monitoring changes in health status, which are needed to optimize formal and informal care as well as apprise medical treatment^18^.

Very few studies have reported monitoring dementia patients in their home using wireless multi-sensor systems^31^, while most of the studies reported in literature were evaluated with elderly subjects using different modalities of sensors^20^. This study adds to the information supplied by our validation study of the sensor devices^21^ and to the classifier performance studies^27^,^32^ published earlier. In addition to the eight clinically relevant ADL often studied^21^,^27^, two ADL (Visitors, Out of home) important for social life and mobility were also detected in this study which is a great step towards reliable ADL monitoring in real life. The results gathered from community-dwelling healthy adults and patients are first of its kind and can be translated to other domicile types.

For statistical and clinical validation, it is important to discriminate the ADL behaviour between healthy controls and patients using quantitative values. Our results show that PP techniques which have already been established to study heart rate variability in other diseases^28^,^33^ can also be successfully applied to distinguish the variability of ADL performance. The high variability in ADL performance in dementia patients may be attributed to the influence of cognition on ADL^34^, as patients may tend to start an activity, interrupt the current activity and start another one, finally losing track of activities and their completeness, leading to dissolving patterns resulting in chaotic daily routines. The speciality of the PP technique is that it can be applied to any subset of temporal interval (as long as the data is continuous or repetitive) allowing different visualisation and quantification of the data.

Studies in the neuropsychological field have shown that it is possible to distinguish between healthy and cognitively impaired subjects based on differences in their behavioural patterns^35^. Our activity maps which visualize the ADL behaviour of the participants in a qualitative way revealing abnormal behaviours over a time period support these findings. The structured pattern observed in the healthy controls compared to non-structured routine in the dementia patients are in line with findings reported by Volicer *et al*.^2^ and Paavilainen *et al*.^36^. Trend analysis would facilitate early identification of abnormal behaviour patterns which can be valuable to clinicians to make decisions or diagnoses or send reminders to the patient if required. In addition, these anomalous behaviour patterns can also be used by machine learning algorithms to train cognitive models.

To date, the functional capability of dementia patients is traditionally assessed using questionnaires in a brief clinic visit. In contrast, sensor-based measurement systems allow to continuously monitor patients in their natural environment, provide ecologically valid feedback on their functional capacity and also offers an opportunity to gain valuable and fine-grained information (e.g.: time and duration of ADL, cognitive status, medication-related improvements, physical ability, sleep problems, disrupted circadian rhythms and emotional state of the patients) that cannot be obtained through paper and pencil measures. The large pool of monitoring data can give rise to finger prints of patient behaviour, trends and anomalies^37^. The deep data mining techniques applied to the sensor data has great potential for a preventive approach in healthcare services. Sensor based systems provide longer and reliable assessment and are ideal for patients living on their own. Owing to their discreet approach, the sensor system also maintains a high level of privacy. In a recent sensor based study on multi-domain mild cognitive impairment (MCI) and amnestic MCI patients, Seeyle *et al*.^38^ showed significant correlation between the cognitive health status of the subject and the level of assistance required to complete a transcripted ADL. Similarly, abnormal behaviours in ADL can be related to the cognitive status of the patient.

The average CDR (1.2 ± 0.4) of the patient population in our study supports the discrimination in ADL performance between healthy and moderate to severe dementia patients. The discriminatory power for early dementia patients or early transition to MCI is questionable due to the subtle and small changes in ADL patterns. The best approach, irrespective of the outcome of future studies, might be a cutoff derived in concurrence with the clinical values such as MMSE^39^, CERAD^40^, CDR^41^ etc. Studies with patients at different levels of cognitive impairment are thus required to detect the sensitivity of the discriminatory power of the recognition system. Increasing accuracy, specificity and sensitivity with increasing duration of measurement, justify further longitudinal measurement studies to check the face-validity and reliability of the discrimination between healthy controls and dementia patients. Moreover, longitudinal studies with huge amount of data for training the ground truth can make way for trends of a small magnitude to be detectable. Future work should also address the case of multi-inhabitants and outdoor activities.

In summary, unobtrusive sensor-based recognition systems are economical, reliable and promising technique for detecting decline in cognitive abilities via ADL monitoring. It has the potential to provide doctors and patients with tools to predict and detect changes in health status. Longitudinal and follow-up studies are required to study ADL patterns which can be potential surrogate markers of a person’s dementia and to be able to predict clinically significant changes in the course of the patient’s disease. Furthermore, these systems provide an additional value for families who require information regarding the need for support within the home to facilitate the independence of their family member^14^. Certain environmental modifications may be necessary and will need to be studied in detail to make this possible in the future. To surmise, sensor-based recognition systems over the long run can help to make “aging in place” a possibility for elderly people and patients suffering from dementia.

## Methods

### Study design and participants

We conducted an observational study with ten dementia patients recruited via the Memory Clinic of the University Hospital of Old Age Psychiatry, Bern, Switzerland and ten healthy controls recruited via advertisement in the Senior University of Bern. The study was carried in accordance with the current version of the Declaration of Helsinki and approved by the Ethics Committee of the Canton of Bern, Switzerland. All procedures related to the study were explained to the participants and a written informed consent was obtained prior to participation. No compensation for participation was provided. All data collected were anonymized at source.

Probable dementia was diagnosed either using ICD-10^42^ or according to the guidelines set by the National Institute of Neurological and Communicative Disorders and Stroke and the Alzheimer’s Disease and Related Disorders Association criteria^43^. All patients were assessed with the standardized CERAD test battery^40^, the informant-based Barthel ADL score (BADLS)^10^ and the clinical dementia rating (CDR)^41^. All patients and healthy controls on inclusion underwent the Mini-Mental State Examination (MMSE)^39^, Clock drawing Test (CDT)^44^, the Trail Making Test A and B (TMT-A, TMT-B)^45^ and the “Timed Up & Go” Test^46^ to assess cognition and mobility.

The main inclusion criteria were age > 60 years and living alone in community. In addition, the inclusion criteria for healthy controls were no cognitive impairment (MMSE score > 26), or no significant motor impairment (“timed Up & Go” Test < 12 s). Patients and healthy controls were age- and gender- matched, while the cognitive and executive parameters differed significantly between the two groups (Table 2).

### Sensor-based measurement system

**Figure 4:**
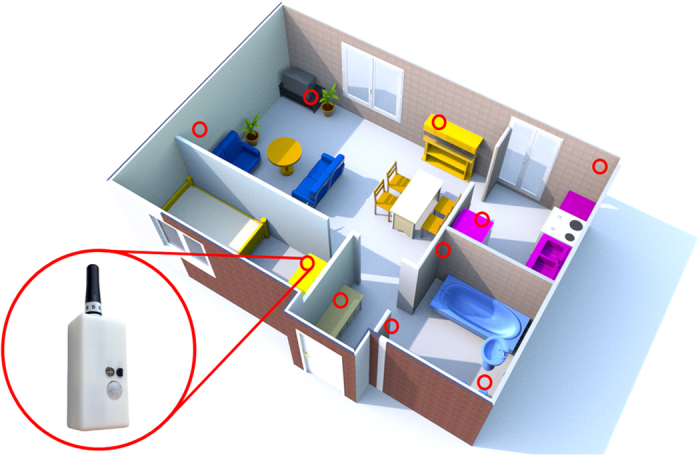
Floor plan of an apartment showing placement of sensor boxes (red circle). Each sensor box (inset photo) captures light, temperature, humidity, movement and acceleration. The floor plan was created using Sweet Home 3D version 5.2a. *Sweet Home 3D, Copyright (c) 2005–2016 Emmanuel PUYBARET/eTeks* <  info@eteks.com* >. *

An unobtrusive sensor network comprising of ten wireless sensor boxes and a central computer unit (CCU)^21^ were installed in the home of the participants. The sensor boxes, each (*l* × *w* × *h* = 15 mm × 30 mm × 60 mm, weight = 80 g) comprising of five sensors (temperature [°C] (DS18B20, Dallas Inc.), humidity [g/m3] (SHT21P, SENSIRION), luminescence [lx] (AMS302, Panasonic Inc.), presence [V] (passive infrared radiation EKMB1101111, Panasonic Inc.), acceleration [m/s2] (ADXL345, Analog Device) capture ambient data with a sampling frequency of 1/5 Hz and transmits them to the CCU using a wireless protocol. The sensor boxes were installed at a height of 1.8–2.2 m such that there was no obstruction of view, facing towards the middle of the room as illustrated in Fig. 4. Additional sensor boxes were placed in the kitchen (in the fridge door) and bathroom (on the flush handle). Once set up and initialized, the sensor boxes recorded around 9,600 person-hours of the five ambient sensor values autonomously and continuously for twenty consecutive days.

### Data analysis

Eight ADL namely “Sleeping”, “Grooming”, “Toileting”, “Getting ready for bed”, “Cooking”, “Eating”, “Watching TV” and “Seated activity”^21^ and two socially important activities, namely “Out of home” and “Visitors”, were defined for recognition. The eight ADL consisting of both basic ADL and IADL may require certain degree of cognitive ability for execution and were used to discriminate between healthy controls and dementia patients based on their ADL performance. The two ADL “Out of home” and “Visitors” are important indicators of mobility and social life. Their recognition was just informative to give completeness to the activity pattern of the participants and not of primary interest in this study.

As a pre-analysis step, the ambient data was sorted room-based, followed by a chronological sort of the individual room-related data^21^. An in-house developed CAR classifier^27^, built on the idea of pattern recognition, was applied to detect and recognize ADL from the sorted ambient data. The ADL recognition within the CAR classifier is based on the assumption that irrespective of the daily routine and cognitive status of the participants, specific patterns with specific duration and timing occur every day. The CAR classifier validated in a prior study (sensitivity 94.36% and specificity of 98.17%)^27^ does not require training data and was applied in the same manner for ADL recognition in this study.

The recognized ADL were then plotted against the time period of twenty days to generate an activity map^27^. The activity map is a temporal representation of the recognised ADL over the complete measurement duration and helps to visualize the behaviour patterns of the participants by introducing colour codes for each ADL. The recognised ADL are stored as lines of time series to build a list. This list is plotted such that the x-axis is the day of measurement and the y-axis displays the time and duration of the corresponding ADL. Thus for each subject, the activity map consisted of colour coded ADL arranged according to the point of ADL initiation and its duration in time.

The difference in the ADL performed by the healthy controls and patients were quantified using the Poincaré plot (PP)^29^. Poincaré theorem is a mathematical tool developed by Jules Henri Poincaré (1854–1912) for analysing complex systems. Classical PP graph the value of one data point of the original time series against the next and can be used to distinguish chaos from randomness. We apply this technique to analyse an entire recording of a subject at once, such that the PP is a scatter plot of the current ADL (ADL_time_) plotted against the preceding recognised ADL (ADL_time – Δtime,_ where Δtime = 24 hours). The PP technique summarizes the data, at the same time allowing to extract the information on short- and long-time behaviour of the participants^28^. Delays of 24 hours are of special interest to us, as it allows us to analyse how activity patterns repeat themselves from day to day.

The PP for a subject’s ADL data vector (equation (1)), composed of recognised ADL data points (*s*_*i*_) and length N (number of recognised ADL datasets over the complete measurement duration)


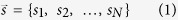


consists of ordered pairs (equation (2)) such that each point in the plot corresponds to two adjacent points (equation (3), (equation (4)).


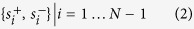



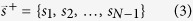



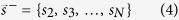


The PP technique was applied on the recognised eight ADL related datasets from dementia patients and healthy controls, whereby elongated cloud of points oriented along a line of identity were obtained (Fig. 2). In case of close resemblance of day-to-day activity patterns, the data points tend to concentrate in the vicinity of the identity line. The quantitative analysis of PP by means of mathematical characterization of the shape of the plot (Ellipse-Fitting technique) introduces various descriptors (e.g.: centroid, long/major axis, short/minor axis) which quantifies the information contained within the plot^47^. The PP centroid is the center of mass of the ellipse and lies on the line of identity (y = x) where the major axis intersects the minor axis. The centroid, long axis, and short axis of the PP were calculated for each participant and each ADL to condense the information of the plot to three independent quantities^48^. Of the three PP quantities, the centroid was selected to depict the heterogeneity in ADL performance.

To quantify the discriminative power of the ADL recognition system the accuracy, sensitivity and specificity were analysed over time. With the aid of the ROC analysis^30^, an optimal cutoff to distinguish healthy subjects from dementia patients, was calculated. ROC give us the ability to assess the performance of the classifier over its entire operating range. An optimal cutoff or threshold needs to be found, such that there are minimum false diagnoses, finding a tradeoff between specificity and sensitivity. To find the optimal cutoff, the mean heterogeneity defined by the centroid of the PP was calculated for each individual over 20 days and averaged for the two groups. The final cutoff value to distinguish between healthy controls and dementia patients resulted from the arithmetical mean of the average heterogeneity (PP centroids) of the two groups. The cutoff was calculated dynamically with every additional dataset within each fold of the cross-validation. Based on this cutoff the discriminative power was analysed with the aid of a leave-one-out cross validation. The function was trained on all the data except for one data point and a prediction (healthy subject or dementia patient) was made for that point. The rate of false positives (FP), false negatives (FN), true positives (TP), and true negatives (TN) was computed, which was then used to calculate the sensitivity (equation (5)), specificity (equation (6)) and accuracy (equation (7)) of the system over the 20 consecutive days of measurement. Sensitivity is the proportion of TP that are correctly identified, while specificity is the proportion of TN that are correctly identified by the system.


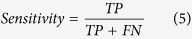



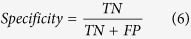



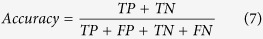


All algorithms were implemented in Matlab R2007b (The MathWorks, Inc.).

### Statistical analysis

Statistical analysis was performed using the Statistical Package for Social Sciences (SPSS Version 20, IBM). Normal distribution of data was examined using Q-Q plots. Means and standard deviations (SD) were calculated. Chi-square tests and one-way analysis of variance (ANOVA) were used for the demographics. The heterogeneity in ADL performance (PP centroids) was analyzed using the Mann-Whitney U Test. All reported p-values are two-tailed and a p < 0.05 was considered significant for comparing demographics, while p < 0.01 was considered significant for comparing ADL performance.

## Additional Information

**How to cite this article:** Urwyler, P. *et al*. Cognitive impairment categorized in community-dwelling older adults with and without dementia using in-home sensors that recognise activities of daily living. *Sci. Rep.* **7**, 42084; doi: 10.1038/srep42084 (2017).

**Publisher's note:** Springer Nature remains neutral with regard to jurisdictional claims in published maps and institutional affiliations.

## Figures and Tables

**Table 1:** ADL classification and Poincaré plot quantification.

	*ADL Classification*			*Poincaré plot Centroid*		
	*Total ADL*	*Healthy Controls (n* = *10)*	*Dementia Patients (n* = *10)*	*Healthy Controls (n* = *10)*	*Dementia Patients (n* = *10)*	*p*
		*n* = 10	*n* = 10	*n* = 10	*n* = 10	
Sleeping	512	234	278	55.28 ± 3.9	90.33 ± 11.5	0.009
Grooming	395	211	184	49.33 ± 3.3	84.52 ± 14.6	0.028
Toileting	614	276	338	59.10 ± 6.0	100.03 ± 10.4	0.002
Getting ready for bed	387	208	179	55.27 ± 3.2	104.94 ± 13.3	0.001
Cooking	408	221	187	54.35 ± 4.4	106.6 ± 9.5	0.001
Eating	548	231	317	54.73 ± 3.8	103.6 ± 16.7	0.028
Watching TV	644	317	327	47.03 ± 2.3	77.92 ± 7.3	0.003
Seating activity	342	162	180	58.75 ± 4.5	105.84 ± 8.1	0.001
Visitors	416	85	331	n.a.	n.a.	n.a.
Out of home	296	152	144	n.a.	n.a.	n.a.
Total	4562	2097	2465			

Data are mean ± standard error of mean.

ADL = activity of daily living.

Statistical Test: Mann-Whitney U Test, *p* < 0.01 significant; n.a = non-applicable.

**Table 2:** Clinical and demographic characteristics (*n* = 20).

	Healthy Controls	Dementia Patients	Statistic	*p*
	*n* = 10	*n* = 10		
Age (years)	73.9 ± 6.7	76.7 ± 8.2	F = 0.687	0.537
Gender (% male)	30	30	χ^2^ = 0.000	1.000*
MMSE [max = 30]	29.1 ± 1.1	23.0 ± 5.1	F = 8.127	0.012
CDT [max = 9]	9.0 ± 0.0	4.5 ± 2.8	F = 4.366	0.050
TMT-A (sec)	39.1 ± 20.0	73.6 ± 7.9	F = 4.221	0.056
TMT-B (sec)	62.6 ± 32.3	178.0 ± 45.6	F = 4.662	0.045
BADL [max = 100]	n.a.	94.5 ± 2.1	n.a.	n.a.
CDR	n.a.	1.2 ± 0.4	n.a.	n.a.

Data are mean ± standard deviation or %. Statistical tests: ANOVA, *Chi-Square tests; MMSE = Mini-Mental State Examination, CDT = Clock Drawing Test, TMT = Trial Making Test, BADL = Barthel Activity of Daily Living, CDR = Clinical Dementia Rating; n.a. = not available.
